# An Analysis of Water Collection Labor among Women and Children in 24 Sub-Saharan African Countries

**DOI:** 10.1371/journal.pone.0155981

**Published:** 2016-06-01

**Authors:** Jay P. Graham, Mitsuaki Hirai, Seung-Sup Kim

**Affiliations:** 1 Department of Environmental and Occupational Health and Department of Global Health, Milken Institute School of Public Health at George Washington University, Washington, DC, United States of America; 2 Department of Global Health, Milken Institute School of Public Health at George Washington University, Washington, DC, United States of America; 3 Department of Public Health Sciences, Korea University, Seoul, South Korea; Indiana University, UNITED STATES

## Abstract

**Background:**

It is estimated that more than two-thirds of the population in sub-Saharan Africa (SSA) must leave their home to collect water, putting them at risk for a variety of negative health outcomes. There is little research, however, quantifying who is most affected by long water collection times.

**Objectives:**

This study aims to a) describe gender differences in water collection labor among both adults and children (< 15 years of age) in the households (HHs) that report spending more than 30 minutes collecting water, disaggregated by urban and rural residence; and b) estimate the absolute number of adults and children affected by water collection times greater than 30 minutes in 24 SSA countries.

**Methods:**

We analyzed data from the Demographic Health Survey (DHS) and the Multiple Indicator Cluster Survey (MICS) (2005–2012) to describe water collection labor in 24 SSA countries.

**Results:**

Among households spending more than 30 minutes collecting water, adult females were the primary collectors of water across all 24 countries, ranging from 46% in Liberia (17,412 HHs) to 90% in Cote d’Ivoire (224,808 HHs). Across all countries, female children were more likely to be responsible for water collection than male children (62% vs. 38%, respectively). Six countries had more than 100,000 households (HHs) where children were reported to be responsible for water collection (greater than 30 minutes): Burundi (181,702 HHs), Cameroon (154,453 HHs), Ethiopia (1,321,424 HHs), Mozambique (129,544 HHs), Niger (171,305 HHs), and Nigeria (1,045,647 HHs).

**Conclusion:**

In the 24 SSA countries studied, an estimated 3.36 million children and 13.54 million adult females were responsible for water collection in households with collection times greater than 30 minutes. We suggest that accessibility to water, water collection by children, and gender ratios for water collection, especially when collection times are great, should be considered as key indicators for measuring progress in the water, sanitation and hygiene sector.

## Introduction

In 2012, the world met the water component of Millennium Development Goal (MDG) target 7c, after reducing by half the proportion of people without access to improved sources of water. Many households now report to be obtaining drinking water from a “protected source”, such as boreholes, protected springs and piped water. Empirical evidence suggests that these protected sources provide higher quality water, and studies have shown that these sources are associated with reduced child morbidity [1,2]. Despite this progress, an important aspect of access–time spent collecting water–has remained largely unaddressed and unexamined as part of MDG target 7c. Time spent collecting water has a variety of potential negative health impacts, especially for women and children.

Over two-thirds of the population in sub-Saharan Africa report leaving their homes to collect water [3], and many rural water systems are often non-functional, exacerbating the difficulty of water collection and augmenting health problems [4]. Thus, the provision of water sources in proximity to households would likely facilitate the collection sufficient water for uses that would improve domestic and personal hygiene. Howard and Bartram suggested that the total water collection time should be less than 30 minutes for basic access levels. Furthermore, they highlighted that water consumption cannot be assured and that hygiene is not possible, unless practiced at the source [5].

Cairncross et al. (1987) highlighted that villagers in a study community without a close water supply stated that they often “cooked little, and only once a day, because of the lack of water”. In the same study, the researchers found that the community with better water access had a prevalence of trachoma of 19% versus 38% in the community without ready access to water [6]. The authors concluded that hygiene practices and hygiene-related health outcomes appeared directly related to how far the water source is from a household. Time spent collecting water has also been found to be associated with significant “coping costs” [7].

Water collection labor can negatively affect human health [8], yet a limited number of studies have been conducted on the detrimental effects of carrying water on household members, including fatigue, musculoskeletal damage and early degenerative bone and soft tissue damage [9]. Water transport can take considerable time and energy, placing high demands on the metabolism, and result in pressure on the skeletal system leading to early arthritis [9]. The most commonly reported adverse effect among the 39 water transporters in South Africa was spinal pain, at 69% [10]. People who carry water may be more prone to injury in rural areas due to higher rates of poverty, chronic malnutrition, and poor health [9].

Furthermore, there may be infectious disease risks associated with collecting water–mainly water-based diseases contracted through contact with water containing infectious microorganisms (e.g., Shistosomes). Qualitative research has found that many children do not want to report diseases that they contracted from carrying water due to the social stigma associated with them, such as worm infections [10]. The same study found that many of the children involved in the study associated a direct relationship between how many trips per day they spent collecting water and their health–more trips per day was worse for their health [10]. A meta-analysis of six studies assessing the relationship between the incidence of diarrhea and water carrying suggests that distance from water supply is an important risk factor for diarrheal disease in children [11]. An analysis of cross-sectional data showed that a five-minute decrease in the time to a water source was associated with a 14% drop in diarrhea risk and a higher bodyweight score in children under five. A fifteen-minute decrease in collection time was associated with a 41% decrease in diarrhea risk for the same age group. The authors noted that this level of reduction in diarrheal disease morbidity is on par with reductions associated with sanitation, hand washing, and water disinfection interventions [12]. More recently, a case-control study in Kenya found that water collection time greater than 30 minutes was associated with an increased risk of moderate-to-severe diarrhea among children, which supports the use of 30 minutes as a metric in the United Nations’ definition of an improved water source [13].

### Why a gender- and child- perspective for water collection labor is needed

In Sub-Saharan Africa (SSA), water collection labor needs to be considered from a gender- and child- perspective for many reasons. First, women and children are generally responsible for water collection in many SSA countries. Geere et al. reported that the most commonly observed water carriers in six rural communities of South Africa were adult women (56%) followed by female children (31%), male children (10%), and adult males (3%) [10]. Sorenson et al. examined water transport by women and children in 15 sub-Saharan countries and showed that women were the primary collectors of water in over half of the households included in the study [14]. Only two of the fifteen countries, Cameroon and Nigeria, had less than half of the households reporting women as their primary water collector at 45.8% and 46.6%, respectively [14]. The range of children being reported as the primary water collector for the household among the same 15 countries ranged from the lowest in Guinea-Bissau at 5% to the highest in Burundi at 39.4% [14].

Second, women's lives are strongly influenced by water collection labor in SSA countries. Cairncross et al. noted, “The shorter time spent in water collection by the women of Namaua permits them 48 minutes more rest per day–almost half the time saved. Much of the time spent resting by the women is passed in the company of their children; it is also available, where facilities are present, for educational, community or productive activities” [6]. Risk of sexual violence, as well as the stress associated with that risk, may also be increased when women and girls must travel long distances away from home to collect water [15].

Third, water collection labor can negatively influence children's schooling in SSA countries.

A participatory observational study in South Africa reported that children spend an average of 19.5 hours in domestic activities where transporting water took up the majority of time, followed by solid fuel collection, and housekeeping [16]. The study also reported that due to water collection activities, children often left school early and experienced fatigue and difficulties in concentrating on their studies [16]. Another study found that older children may be pulled out of school to watch younger children while mothers go to collect water or to collect the water themselves [17]. When asked about their perceptions of domestic chores, including fetching water for their household, nearly all young girls in Ethiopia felt that it limited their ability to partake and succeed in school [18]. Porter et al. also found that children had to transport goods and water to pay for school fees. The authors noted that one-third of girls and boys in Ghana, and 8% of girls and 3% of boys in Malawi, reported to arrive late for school because of water collecting activities [19].

Fourth, women and children generally have less physical capacity to carry heavy loads in contrast to adult men [12]. A recent study suggested that children experience extreme pain from water collection activities, and the level of pain and fatigue may be determined by the distance they travel [10]. Some children, however, expressed positive feelings about water collection chores because they can help their family meet basic needs, get exercise, and earn money by delivering water to households [10].

Given the large number of people affected by water collection labor in sub-Saharan Africa, the present study aims to more fully describe water collection practices among all sub-Saharan African countries with available data. The objectives of this study are to: 1) estimate the absolute number of people affected by long water collection times (i.e. greater than 30 minutes), 2) characterize gender differences in water collection labor among adults in the households that report spending more than 30 minutes collecting water, and 3) elaborate on gender differences in water collection labor among children in the households that report spending more than 30 minutes collecting water.

## Methods

Publicly available data were collected on household water collection from two major international survey programs, the Multiple Indicator Cluster Surveys (MICS) conducted by country governments in collaboration with UNICEF, and the Demographic and Health Survey (DHS) conducted by the ICF International and funded by the United States Agency for International Development (USAID). The MICS and DHS typically contain a sample size ranging from 5,000 to 30,000 households, varying in accordance with country size, and are conducted every three to five years.

This analysis included the Sub-Saharan African countries that had conducted DHS or MICS household surveys between 2005 and 2012. If two or more surveys were available for one country, the most recent was used to obtain the most current estimate. DHS and MICS surveys were used for the analysis if they had data for the following questions: 1) “What is the main source of drinking water for members of your household?”; 2) “Where is that water source located?”; 3) “How long does it take to go there, get water, and come back?”; and 4) “Who usually goes to this source to fetch the water for your household?”, with sub-questions, “Is this person under age 15?” and “What sex?”. Options to answer the first question include: “In own dwelling”, “In own yard/plot”, and “Elsewhere”. The third question provided a blank line followed by the word “Minutes” and provides the option “Don’t know”. The fourth question provides the options “Adult woman (≥ 15 years of age)”, “Adult male (≥ 15 years of age)”, “Female child (<15 years of age)”, and “Male child (<15 years of age)”. This last set of questions was used to calculate the proportion of households where children, male or female, are primarily responsible for water collection. It should be noted that for estimating the time spent collecting water, the DHS and MICS questions do not determine whether households are spending this time waiting for water (e.g., queuing at a water point) or walking. Furthermore, the questions do not determine how many trips a household may make in one day. Households were disaggregated by urban or rural place of residence, a variable included in the DHS and MICS surveys.

We calculated the absolute number of adult females and children affected by water collection times greater than 30 minutes in 24 SSA countries by using information from the World Bank Database and DHS or MICS dataset. For example, in urban areas of Burkina Faso, we estimated the absolute number of children with water collection times greater than 30 minutes by using the following formula: (A/B)*C*D*E. Data for (A) was derived from the World Bank Database, and data for (B,C,D,and E) were from DHS or MICS.

- (A) The total population in Burkina Faso (13.82 million) * The proportion of urban population in Burkina Faso (29%)
- (B) The average number of people in urban households in Burkina Faso (5.1 people per household)
- (C) The proportion of urban households without water on premises in Burkina Faso (68%)
- (D) The proportion of urban households which reported water collection labor (>30 min) among C (22%)
- (E) The proportion of urban households which reported children as the primary water collector among D (25%)"

The DHS and MICS are stratified, clustered, and two-stage surveys and are designed to be representative of the country as a whole, and in combination with population estimates (World Bank data) and urban and rural residence, available as part of DHS and MICS, were used to estimate urban and rural water collection practices. In most cases, there is over or under sampling in order to expand or reduce the number of cases for certain areas or sub-populations. The household weights provided in the datasets were applied in this analysis by using the Stata svyset and svy commands to get representative results of the entire population. Because water collection practices vary for urban and rural settings, this report provides tables disaggregated for urban and rural areas. We downloaded DHS and MICS datasets from www.measuredhs.com and http://mics.unicef.org/surveys, respectively. World Bank data were available at: http://data.worldbank.org. The data analysis was conducted using STATA SE© version 10 (STATA Corp., College Station, TX).

## Results

### Water Collection in 24 SSA countries

There were 24 sub-Saharan African countries that had DHS or MICS data available on the questions of interest. These countries varied significantly in terms of population size (0.2 million to 178.5 million), proportion of urban/rural residents (35% to 88% rural), per capita GDP ($604 to $9,583), the annual GDP growth rate (–36% to +11.3%) and the percentage of the population with access to improved water supplies, as defined by the WHO/UNICEF Joint Monitoring Programme (32% to 97%) (Table 1).

**Table 1:** Demographic characteristics of the 24 countries included in the analysis.

Country	Population^1^ (Millions)	Urban/Rural^2^ (%)	Per capita GDP^3^ (USD)	Annual change in GDP^4^ (%)	Access to Improved Water Supply^5^ (%)
Burkina Faso	17.4	29/71	1,514	6.5	82
Burundi	10.5	12/88	772	4.6	75
Cameroon	22.8	54/46	2,830	5.6	74
Central African Rep.	4.7	40/60	604	-36.0	68
Cote d’Ivoire	20.8	53/47	3,210	8.7	80
Ethiopia	96.5	19/81	1,380	10.5	52
Gambia	1.9	59/41	1,661	4.8	90
Ghana	26.4	53/47	3,992	7.6	87
Guinea	12.0	37/63	1,253	2.3	75
Lesotho	2.1	27/73	2,576	5.5	81
Liberia	4.4	49/51	878	11.3	75
Madagascar	23.6	34/66	1,414	2.4	50
Malawi	16.8	16/84	780	5.0	85
Mali	15.8	39/61	1,642	2.1	67
Mauritania	4.0	59/41	3,043	6.7	50
Mozambique	26.5	32/68	1,105	7.4	49
Namibia	2.3	46/54	9,583	5.1	92
Niger	18.5	18/82	916	4.1	52
Nigeria	178.5	47/53	5,602	5.4	64
Sao Tome & Principe	0.2	65/35	2,971	4.0	97
Sierra Leone	6.2	40/60	1,544	5.5	60
Somalia	10.8	39/61	ND^6^	ND^6^	32
Swaziland	1.3	21/79	6,685	2.8	74
Zimbabwe	14.6	33/67	1,832	4.5	80

^1^ World Bank. (2015). World Bank. (2015). Population, total. Available at: http://data.worldbank.org/indicator/SP.POP.TOTL.

^2^ World Bank. (2015). Urban population (% of total). Available at: http://data.worldbank.org/indicator/SP.URB.TOTL.IN.ZS.

^2^ World Bank. (2015). Rural population (% of total population). Available at: http://data.worldbank.org/indicator/SP.RUR.TOTL.ZS.

^3^ World Bank. (2015). GDP per capita. Available at: http://data.worldbank.org/indicator/NY.GDP.PCAP.CD.

^4^ World Bank. (2015). GDP growth (annual %). Available at: http://data.worldbank.org/indicator/NY.GDP.MKTP.KD.ZG/countries.

^5^ World Bank. (2015). Improved water supply (% of population with access to a source of water that is considered protected) Data for access to improved water supply are from 2012 except for Somalia, using 2011 data. Available at: http://data.worldbank.org/indicator/SH.H2O.SAFE.ZS.

^6^ No Data.

### Water on Premises

There was also considerable heterogeneity of water-related characteristics among the rural and urban areas of the countries (Table 2). In rural areas, the proportion of households lacking access to water on their premises was more than 90% in half of the countries (13 countries). Only four countries had less than 75% of rural households without access to water on their premises (68%, Cote d’Ivoire; 68%, Namibia; 60%, Mali; and 73%, Swaziland). For urban areas, access to water on the household premises was higher–only two countries, Central African Republic (89%) and Liberia (85%) had more than 75% of households lacking water on household premises. Fourteen countries had between 50% and 75% of households lacking water on their premises and eight had less than half of households without water on their premises. Typically, countries with low levels of household-level access to water in urban areas also had low coverage levels in rural areas, but this was not the case in Ethiopia, Lesotho, and Swaziland. Ethiopia had 99% of rural households lacking water on their premises versus 50% of urban households, Lesotho had 95% of rural households versus 37% urban, and Zimbabwe 86% rural households versus 23% urban.

**Table 2:** Characteristics of the 24 countries included in the analysis based on Demographic and Health Surveys and Multiple Indicator Cluster Surveys.

Country	Survey	Number of households surveyed	Households without water on premises						Households without water on premise and with > 30 minute water collection^1^					
			Prevalence		Primary water collector				Prevalence		Primary water collector			
			Urban	Rural	Urban		Rural		Urban	Rural	Urban		Rural	
					Adult female	Child	Adult female	Child			Adult female	Child	Adult female	Child
Burkina Faso	MICS 2006	6,034	68%	97%	67%	12%	90%	5%	22%	34%	64%	25%	88%	6%
Burundi	MICS 2005	8,220	57%	98%	59%	32%	57%	36%	22%	36%	60%	32%	57%	34%
Cameroon	MICS 2006	9,856	68%	92%	39%	28%	51%	28%	13%	27%	40%	29%	57%	25%
Central African Republic	MICS 2006	11,941	89%	98%	67%	21%	77%	12%	14%	28%	64%	26%	79%	12%
Cote d'Ivoire	MICS 2006	7,600	15%	68%	80%	12%	87%	9%	8%	24%	89%	9%	90%	6%
Ethiopia	DHS 2011	16,702	50%	99%	70%	12%	72%	20%	22%	43%	72%	18%	71%	20%
Gambia	MICS 2005/06	6,175	45%	88%	76%	8%	85%	9%	17%	14%	82%	8%	90%	6%
Ghana	DHS 2008	11,778	58%	94%	60%	16%	66%	16%	5%	12%	65%	7%	79%	9%
Guinea-Bissau	MICS 2006	5,452	58%	89%	92%	5%	94%	5%	10%	16%	85%	12%	90%	8%
Lesotho	DHS 2009	9,391	37%	95%	68%	8%	72%	9%	10%	18%	77%	5%	77%	9%
Liberia	DHS 2007	6,824	85%	92%	43%	38%	66%	24%	6%	2%	39%	39%	63%	18%
Madagascar	DHS 2008/09	17,857	67%	88%	62%	16%	71%	17%	3%	5%	62%	13%	71%	12%
Malawi	MICS 2006	31,200	72%	98%	83%	6%	88%	7%	26%	36%	85%	6%	89%	5%
Mali	DHS 2006	12,998	43%	60%	55%	44%	69%	31%	3%	5%	52%	46%	64%	32%
Mauritania	MICS 2007	10,937	19%	77%	50%	12%	75%	9%	39%	58%	51%	12%	75%	9%
Mozambique	MICS 2008	14,300	75%	98%	80%	11%	88%	7%	28%	43%	84%	9%	88%	7%
Namibia	DHS 2006/07	9,200	19%	68%	59%	5%	62%	15%	4%	21%	68%	4%	58%	19%
Niger	DHS 2006	7,660	53%	93%	41%	46%	63%	26%	6%	33%	28%	55%	53%	32%
Nigeria	MICS 2007	28,603	73%	88%	53%	17%	44%	19%	16%	30%	46%	16%	47%	17%
Sao Tome & Principe	DHS 2008/09	3,536	59%	75%	73%	12%	67%	13%	22%	16%	69%	10%	71%	11%
Sierra Leone	MICS 2005/06	8,000	74%	98%	45%	37%	77%	16%	11%	10%	42%	33%	79%	16%
Somalia	MICS 2006	6,000	61%	99%	51%	7%	72%	6%	30%	52%	49%	4%	63%	6%
Swaziland	DHS 2006/07	4,843	24%	73%	63%	7%	70%	14%	7%	27%	67%	18%	72%	11%
Zimbabwe	MICS 2009	12,500	23%	86%	70%	4%	82%	7%	20%	22%	70%	6%	83%	5%

^1^ The denominator used to calculate the percentage is the number of households without water on premises for either urban or rural residence.

### Water collection times greater than 30 minutes

The percentage of households without water on their premises and that reported spending more than 30 minutes collecting water varied significantly (Table 2). In rural Liberia, for example, only 2% of households without water on premises reported spending more than 30 minutes collecting water, while it was 58% in rural Mauritania. In 13 of the 24 countries, 20–50% of rural households reported spending more than 30 minutes collecting water. In nine countries, less than 20% of rural households reported spending more than 30 minutes collecting water. The percentage of urban households that reported to spend more than 30 minutes collecting water was generally lower in contrast to rural households, ranging from 3% in urban Madagascar to a high of 39% in urban Mauritania. Overall, one-third of the countries had less than 10% of their urban households reporting to spend more than 30 minutes collecting water, one-third of countries fell into the 10 to 20% category, and the remaining third had between 20% and 39% of urban households reporting to engage in this time-intensive water collection labor.

### Water collection by adult females

**Fig 1:**
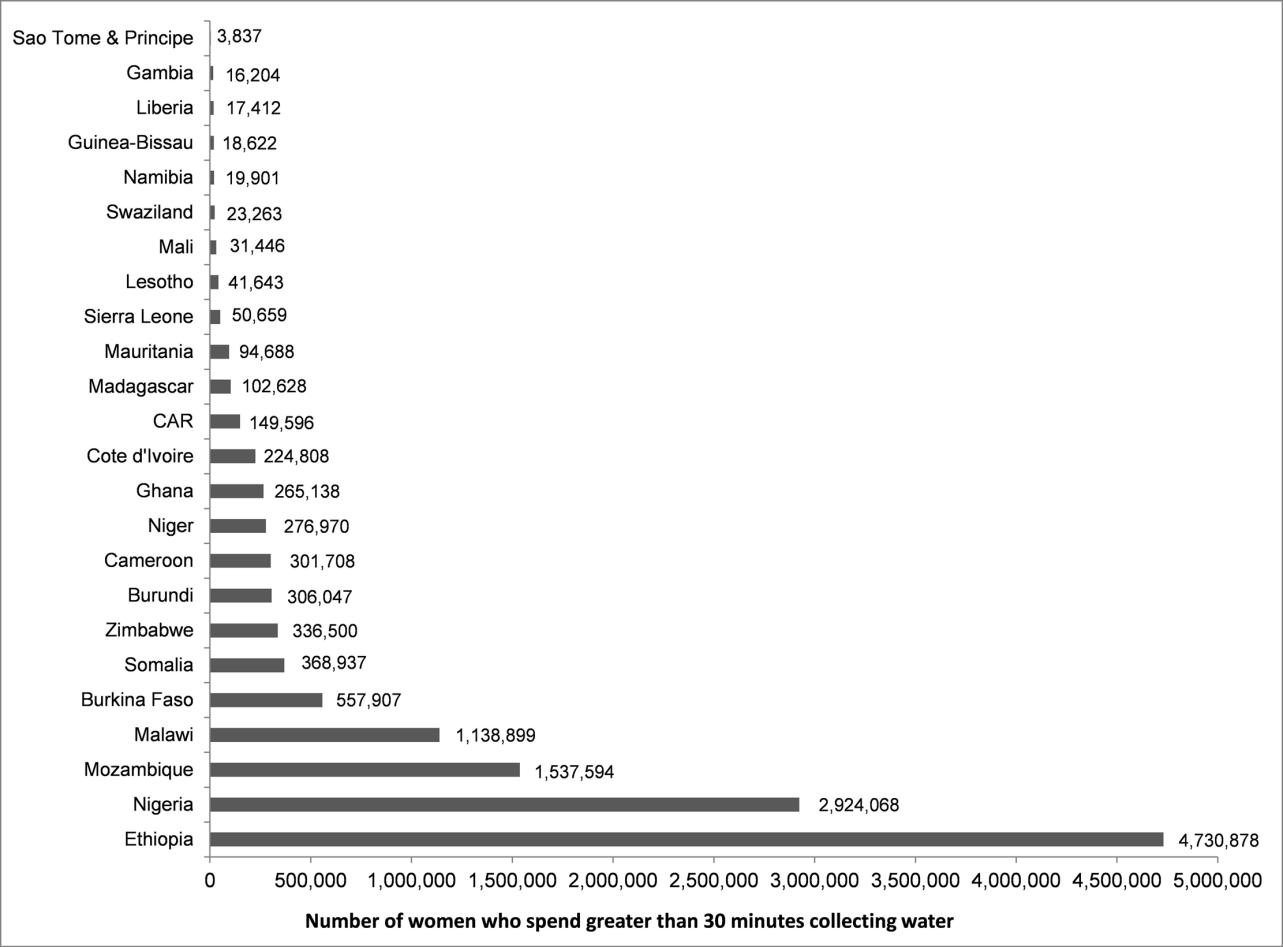
Number of adult females who are reported as the primary collectors of water, by country, for households reporting to spend greater than 30 minutes collecting water.

Across the 24 countries, adult females were predominantly responsible for water collection (Table 2). In 10 countries, adult women were reported to be the primary collector of water in more than three-quarters of households across the country. Liberia was the only country where less than half of water collection (greater than 30 minutes) was done by an adult female (46%). For each country, the prevalence of adult females as the primary collectors of water was generally similar across rural and urban areas, as well as for collection times greater or less than 30 minutes. There were, however, six countries where the prevalence of women as the primary water collectors among all households without water on their premises differed by over twenty percentage points between urban and rural areas. These percentage point differences were observed in rural areas of: Burkina Faso (+23%), Liberia (+23%), Mauritania (+25%), Niger (+22%), Sierra Leone (+32%), and Somalia (+21%). When reported water collection times were greater than 30 minutes, the proportion of adult females as the primary collectors was lower in both urban and rural settings of six countries and higher in six countries. There were four countries (Fig 1) where more than one million households reported an adult female is the primary water collector when water collection times were above 30 minutes: Nigeria (2.9M), Ethiopia (4.7M), Malawi (1.1M), and Mozambique (1.5M). Only 10 of the 24 countries had less than 100,000 households that reported an adult female is the primary water collector when water collection times were above 30 minutes (Fig 1).

There was substantial variability in the gender ratio (i.e. percent females/percent males) of adults who performed water collection across countries. All countries had gender ratios above one, indicating that adult women, more than men, shouldered the responsibility for water collection. While 17 of the 24 had gender ratios below 10, seven countries had gender ratios over 10, including: Burkina Faso (15.1), Cote d’Ivoire (26.2), Gambia (18.6), Guinea (53.9), Malawi (18.1), Mali (19.9), and Mozambique (15.9). In urban areas, adult gender ratios were typically smaller than in rural areas. The gender ratio was lower by more than 10 in urban areas (versus rural areas) for Burkina Faso, Gambia, Guinea, and Malawi. Only in Cote d’Ivoire and Mali, the gender ratio was higher by more than 10 in urban areas versus rural areas.

### Water collection by children

**Fig 2:**
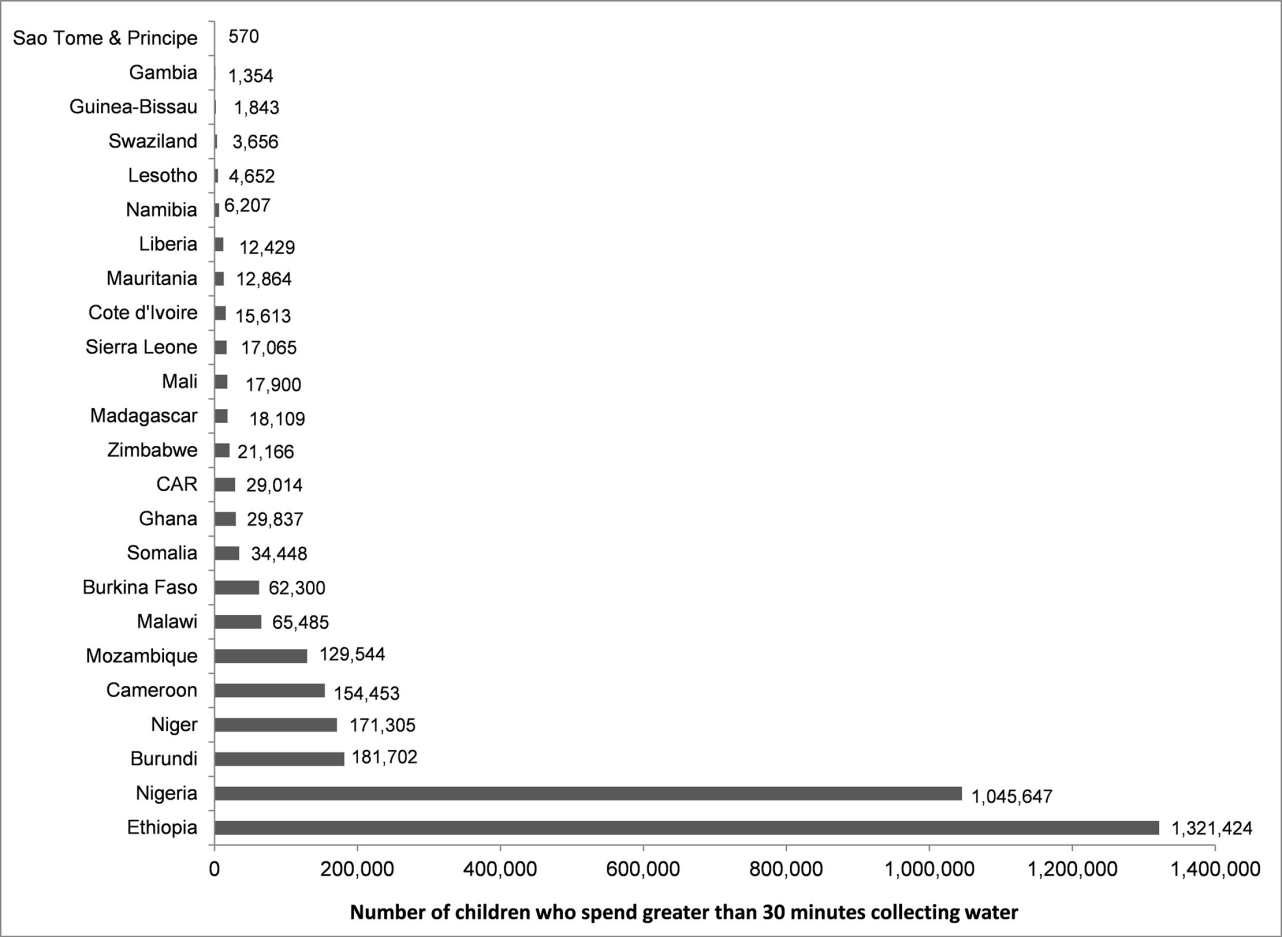
Number of children (<15 years of age) who are reported as the primary collectors of water, by country, for households reporting to spend greater than 30 minutes collecting water.

The percentage of households where children were the primary collectors of water ranged from 4% in urban Zimbabwe to 44% in urban Niger. There was little change in this range when water collection took greater than 30 minutes (Table 1). For urban areas, there were four countries that had greater than 30% of water collection performed by children: Burundi (32%), Liberia (38%), Mali (44%), Niger (46%), and Sierra Leone (37%). For rural areas, there were two countries that had more than 30% of water collection carried out by children: Burundi (36%) and Mali (31%). Water collection by children was more prevalent when collection times went from less than 30 minutes to greater than 30 minutes in urban Burkina Faso (+13%) and urban Swaziland (+11%). In rural areas with household collection times greater than 30 minutes, there were three countries that had more than 30% of water collection by children: Burundi (34%), Mali (34%), and Niger (32%). In urban areas with household collection times greater than 30 minutes, there were five countries that had more than 30% of water collection by children: Burundi (32%), Liberia (39%), Mali (46%), Niger (55%), and Sierra Leone (33%) (S1 Appendix. Summary statistics for countries). There were two countries (Fig 2) where more than one million households reported a child as the primary water collector when water collection times were above 30 minutes: Nigeria (1.0M) and Ethiopia (1.3M). Six of the 24 countries had more than 100,000 households that reported a child as the primary water collector when water collection times were above 30 minutes (Fig 2).

In households where children were reported to be the primary collector of water and where collection times were greater than 30 minutes, female children, versus male children, dominated water collection. There was variability in the gender ratio (i.e. percent females/percent males) of children who performed water collection across countries. Twenty-two countries had gender ratios greater than one indicating that girls, more than boys, shouldered the responsibility for water collection. Most countries had gender ratios below 10; Guinea and Malawi, however, had gender ratios of 12.9 and 10.6, respectively. Two countries had gender ratios of one or below among households where collection times were greater than 30 minutes: Liberia and Niger. There were few instances when the results, by place of residence or by collection times greater than 30 minutes, had children gender ratios below one. In nine countries, gender ratios increased in rural areas, over urban areas, by more than one when collection times were greater than 30 minutes and in three countries the opposite occurred (Table 3).

**Table 3:** Gender ratio (female/male) of adults and children who are reported to be the primary collectors of water for households spending greater than 30 minutes collecting water, by place of residence.

Country	Urban		Rural		Total	
	Adults	Children	Adults	Children	Adults	Children
Burkina Faso	6.5	0.7	18.3	3.4	15.1	1.6
Burundi	7.4	1.3	6.7	1.2	6.7	1.2
Cameroon	1.3	1.3	3.3	1.2	2.5	1.2
Central African Republic	7.2	4.3	8.6	3.6	8.3	3.8
Cote d’Ivoire	66.9	0.4	25.3	3.4	26.2	2.8
Ethiopia	6.5	2.2	8.6	2.9	8.4	2.9
Gambia	10.2	15.2	31.8	7	18.6	9.2
Ghana	2.3	1.4	7.2	1.6	5.4	1.5
Guinea	31.3	5.9	65.1	22.1	53.9	12.9
Lesotho	4.1	1.2	5.3	4.7	5.1	4.2
Liberia	1.8	1.6	3.5	0.7	2.5	1.3
Madagascar	2.5	2.9	4.1	1.9	4	2
Malawi	9.2	7.3	19.7	11	18.1	10.6
Mali	30.8	0.7	18.9	2.1	19.9	1.7
Mauritania	1.6	0.5	4.7	1	4.1	0.9
Mozambique	12.4	3.5	16.9	5.7	15.9	5
Namibia	2.4	1.3	2.5	1.6	2.5	1.5
Niger	1.6	1.3	3.7	1	3.6	1
Nigeria	1.2	1.2	1.3	1.5	1.3	1.3
Sao Tome & Principe	3.3	6.4	4.1	1.3	3.6	2.5
Sierra Leone	1.7	2.5	17.7	2.4	7.5	2.4
Somalia	1	1.4	2.2	1.3	1.9	1.3
Swaziland	4.3	0.1	4.1	2.2	4.1	1.9
Zimbabwe	2.9	0.9	6.7	3.5	6.2	3

## Discussion

This is one of the first studies to describe water collection practices–disaggregated by children versus adults, gender, and urban/rural residence–across 24 sub-Saharan African countries. The results highlight significant variability in water collection practices across countries, but clearly show that female adults and female children consistently carry the greatest responsibility for water collection, for both collection times less and greater than 30 minutes. This analysis builds on the work by Sorenson et al. by providing additional insight into urban and rural differences and gender differences among the children responsible for water collection [14].

There has been a major push since the first water decade (1981–1990) to increase the number of people who can access improved water supplies. It is unclear, however, to what degree improved access affects the time households spend collecting water. This reduction in time could occur when an improved well is placed in proximity to a community [20]. In this study, self-reported access to an “improved water supply”, however, did not appear to be correlated with a high percentage of households having water on their premises. Eighty-seven percent of the surveyed population in Ghana, for example, reported using an improved water source, yet 94% of rural residents and 58% of urban households must travel to collect water. Similarly, in Gambia 90% of its population reported improved water supply, yet 88% of rural residents and 45% of urban residents reported to leave their premises to collect water.

### Limitations

It is important to treat the uncertainties of this study in a transparent manner and highlight its limitations. First, the number of trips is not considered in the two national survey instruments (i.e. DHS and MICS), and it is clear that many households make multiple trips for water collection, which can vary within and across seasons. A study in Haryana, India found that women collected water, on average, 23 times each day during the summer months [21]. Furthermore, there is evidence that households’ ability to recall water collection times is problematic. Davis et al. (2012) studied the effects of a new piped water system introduced in one region of Kenya on the time spent collecting water to another region without such a system [22]. The researchers used both household interviews and GPS devices to estimate water collection times. The data from the GPS units indicated significantly shorter times than the data collected from household interviews. The researchers noted, however, that “the more precise GPS data indicates many short trips under 10 minutes”. Further, they noted that “some users walked 5 minutes to a water point, stayed there for 2 hours, and then walked 5 minutes home” In some cases, the researchers found that households would collect water from more distant sources because it was free of charge [22]. Ho et al. (2014) compared self-reported one-way water collecting time in rural Mozambique against time estimates based on the route distance [23]. The researchers found that self-reported data were poorly correlated with route distance and they noted that more research is needed to determine the best proxy measures for quantifying water collection times [23]. More recently, Nygren et al. found that self-reported water collection times correlated well with GPS coordinate data [13].

This analysis does little for building an understanding of why the countries varied in terms of who in the household was responsible for water collection. Going forward, there is a need to better understand the social dimensions affecting water collection practices. Research has suggested that women’s lack of political power may be an important barrier to gaining better access to water supplies. Researchers have found evidence that the gender of local leaders was associated with investments in water supplies–more investment was made when women were in charge [24]. In some African societies, physically transporting goods, such as water, is considered a lower status activity and deemed more appropriate for females [19]. The same authors considered the differential balance of power in the context of families and communities and how those result in intergenerational gender relations. Mehta (2013) reviews numerous constraints to achieving better water access for women and girls and discusses how women’s participation in decision-making can be advanced [25].

## Conclusion

There is a critical need to reduce the amount of time that women and children spend collecting water. We suggest that accessibility to water, water collection by children, and gender ratios for water collection, especially when collection times are great, should be considered as key indicators for measuring progress in the water, sanitation, and hygiene sector. More work, however, is needed in order to test and potentially improve the validity of questions. For example, the number of trips a household makes per day should be added to national survey instruments to better determine water collection labor.

Longitudinal research studies would be useful to better characterize how improving access to water infrastructure affects households’ opportunity costs, as well as nutritional outcomes and other health outcomes, including road casualties and assaults. Due to the limited research on water transport, current assessments made in the global burden of disease report do not include this as a risk factor in their analysis. If major investments are not made to address water collection labor, the problem could be heightened over time due to the increasing number of people predicted to live in areas with serious water shortages, many of whom are in sub-Saharan Africa.

## Supporting Information

S1 Appendix Summary statistics for countries. (XLSX)
